# A Systematic Review on the Role of SIRT1 in Duchenne Muscular Dystrophy

**DOI:** 10.3390/cells10061380

**Published:** 2021-06-03

**Authors:** Elisa Domi, Malvina Hoxha, Emanuela Prendi, Bruno Zappacosta

**Affiliations:** 1 Department for Chemical-Toxicological and Pharmacological Evaluation of Drugs, Faculty of Pharmacy, Catholic University Our Lady of Good Counsel, Rruga Dritan Hoxha, Tirana 1000, Albania; elisadomi94@yahoo.it (E.D.); b.zappacosta@unizkm.al (B.Z.); 2 Department of Biomedical Sciences, Faculty of Medicine, Catholic University Our Lady of Good Counsel, Rruga Dritan Hoxha, Tirana 1000, Albania; e.prendi@unizkm.al

**Keywords:** Duchenne muscular dystrophy, sirtuin1, inflammation

## Abstract

Duchenne muscular dystrophy (DMD) is a muscular disease characterized by progressive muscle degeneration. Life expectancy is between 30 and 50 years, and death is correlated with cardiac or respiratory complications. Currently, there is no cure, so there is a great interest in new pharmacological targets. Sirtuin1 (SIRT1) seems to be a potential target for DMD. In muscle tissue, SIRT1 exerts anti-inflammatory and antioxidant effects. The aim of this study is to summarize all the findings of in vivo and in vitro literature studies about the potential role of SIRT1 in DMD. A systematic literature search was performed according to PRISMA guidelines. Twenty-three articles satisfied the eligibility criteria. It emerged that SIRT1 inhibition led to muscle fragility, while conversely its activation improved muscle function. Additionally, resveratrol, a SIRT1 activator, has brought beneficial effects to the skeletal, cardiac and respiratory muscles by exerting anti-inflammatory activity that leads to reduced myofiber wasting.

## 1. Introduction

Duchenne muscular dystrophy (DMD) is an X-linked muscular disease with an incidence of 1 in 3500 male births worldwide. It is characterized by dystrophin deficiency due to a mutation in the gene encoding for this protein. Dystrophin is a member of the sarcolemmal dystrophin glycoprotein complex (DGC) which connects the intracellular actin cytoskeleton and the extracellular matrix of skeletal muscle [1,2]. Dystrophin deficiency leads to an alteration of the DGC resulting in a cascade of several events, such as membrane instability, chronic inflammation, muscle degeneration, and myofiber necrosis [1,2,3]. As muscle regeneration processes decrease, muscle fibers are gradually replaced by connective and adipose tissue [4]. Muscle weakness generally begins around the age of three and degenerates until the loss of the ability to walk before the age of thirteen. Unfortunately, life expectancy is between 30 and 50 years and patients usually die because of cardiac and/or respiratory complications [1,5]. Nowadays, there is no cure for DMD, the only approved therapy is the use of glucocorticoids (GCs) such as prednisone, prednisolone and deflazacort, which are able to slow down the muscle decline [6,7]. However, long-term use of these anti-inflammatory agents may cause several side effects. Currently there is an unmet need for innovative therapeutic approaches. Some of the experimental molecular-based strategies, used to modify the DMD gene product, include exone skipping or suppression of premature stop codons. However, these approaches seem to be effective only in a limited number of patients; for this reason, it is necessary to focus on pharmacological strategies applicable in all DMD patients [8,9,10].

Interestingly, several studies have shown that reducing muscular inflammation ameliorates both muscle function and morphology, suggesting that inflammation should be a good therapeutic target [11,12].

Regarding inflammation, an emerging and innovative therapeutic target for DMD seems to be the activation of Sirtuin1 (SIRT1).

SIRT1 belongs to a class of NAD^+^-dependent class III histone/deacetylase proteins, the sirtuins [13]. These enzymes modulate the activity of different nuclear and cytoplasmatic proteins, which in turn regulate several cellular processes [14]. Under stressful conditions, when NAD^+^ levels increase, sirtuin transcription appears to be activated, and this is the reason why these enzymes are considered cell survival mediators. Sirtuins, and especially the SIRT1 isoform, promote longevity by delaying cellular aging, inhibit apoptosis, regulate the cell cycle, the glucose homeostasis and the insulin secretion, and are involved in inflammation, oxidative stress and mitochondrial biogenesis [15,16,17,18].

Due to its role in the physiological processes, SIRT1 plays a key role in various diseases, such as diabetes, obesity, cancer, neurodegenerative and cardiovascular diseases, and age-related pathologies [19].

SIRT1 is found both in the nucleus and in the cytosol, and exerts its action by deacetylating histone and non-histone proteins, such as the tumor suppressor p53, the forkhead box O (FOXO) family of transcription factors, the hypoxia-inducible factor 1-alpha (HIF-1α), the peroxisome proliferator-activated receptor gamma (PPARγ), the cofactor of PPARγ (PGC-1α), Ku70 (a protein involved in DNA repair), and the nuclear factor NF-kB [20,21].

**Figure 1:**
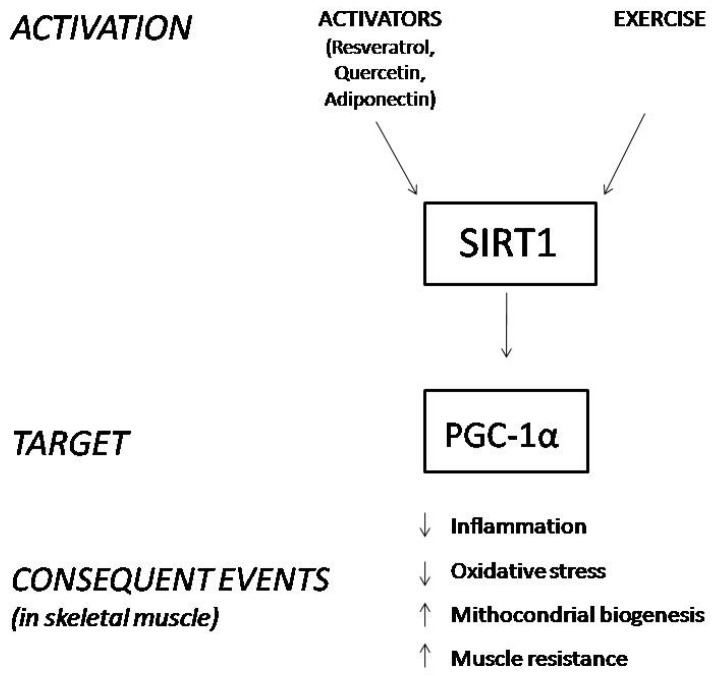
Schematic representation of the consequences derived from SIRT1 stimulation in skeletal muscle.

SIRT1 is expressed in different tissues, and we focus on its role in skeletal muscle, where it deacetylates and activates PGC-1α. The activated form of PGC-1α controls mitochondrial biogenesis and homeostasis, improves muscle resistance, enhances muscle fiber-type switching, and decreases the process of muscle wasting during aging due to its ROS scavenging activity (Figure 1) [22,23,24,25,26,27,28,29]. Hence, the activation of the SIRT1-PGC-1α axis in skeletal muscle ameliorates the phenotype of the X-linked recessive, muscle wasting disease DMD [30].

Since the modulation of SIRT1 leads to several beneficial effects, attention has also been paid to activators of this protein, such as resveratrol and quercetin. Both of them are polyphenols, a class of flavonones that have been largely studied for their anti-inflammatory properties, for their positive effects in reducing oxidative stress and ameliorating cardiac diseases [31,32,33]. Some recent findings suggest that polyphenols may alleviate muscular dystrophic pathologies by activating the SIRT1/PGC-1α axis [34]. Additionally, adiponectin (ApN), a hormone secreted by adipocytes under normal conditions, exerts anti-inflammatory properties in dystrophic muscles by activating the SIRT1-PGC-1α pathway [2,35].

In this systematic review, we aimed to summarize and highlight all the findings concerning the role of SIRT1 as a potential therapeutic target in DMD.

## 2. Materials and Methods

This systematic review was conducted according to the preferred reporting items for systematic review (PRISMA) guidelines [36]. We selected all the necessary data based on the eligibility criteria regarding the role of SIRT1 in Duchenne muscular dystrophy.

### 2.1. Study Design

Nowadays, the elective therapy for Duchenne muscular dystrophy is the use of glucocorticoids, which are unfortunately associated with several side effects. Therefore, we were interested in new possible drug targets that could be exploited for the treatment of this pathology, specifically on a novel hypothetical target, SIRT1, a protein belonging to the sirtuin family. The aim of our research was to collect the information currently available regarding the role of the SIRT1 enzyme in improving the pathophysiology of Duchenne muscular dystrophy, including the effects that its activation brings to skeletal muscles. As the main cause of death of DMD patients appears to be cardiac and/or respiratory failure, we also evaluated the studies concerning the action of SIRT1 in cardiac and respiratory tissues of dystrophic animal models.

### 2.2. Eligibility Criteria

Initially, we defined the eligibility criteria for the inclusion of the studies in this systematic review by analyzing all the original research papers currently present in different databases, describing the correlation between SIRT1 and DMD. Since the activation of SIRT1 is involved in cell survival processes, we wondered how this protein and its activators can slow down the normal degeneration of the muscle tissue in DMD, so we studied the possible mechanism behind SIRT1-mediated muscle regeneration. We extrapolated data concerning the composition of muscle fibers, myofiber damage, force resistance and fatigue resistance. SIRT1 is also involved in inflammatory and oxidative processes, so we investigated the metabolites involved, as well as the mitochondrial activity and damage. Due to cardiomyopathy, the main cause of death in DMD is heart failure, so we searched for the benefits of SIRT1 activation and its activators in dystrophic hearts and their role as cardioprotective agents. Considering that respiratory failure is another primary cause of death in patients with muscular dystrophies, we also assessed the effects of SIRT1 activators in respiratory function.

We included and considered eligible all the studies (in vivo and in vitro) carried out in dystrophic animal models, or in cell cultures of dystrophic animals and humans, which:

- evaluated the expression of SIRT1 in muscle, cardiac and respiratory tissues of dystrophic models;
- demonstrated the effect of SIRT1 activation in the mentioned tissues;
- evaluated the effect of SIRT1 activators in Duchenne muscular dystrophy;
- explained the potential mechanisms of action of SIRT1 in improving the pathophysiology of DMD.

We excluded and considered as non-eligible all articles that, although concerning potential new therapies for Duchenne muscular dystrophy, did not include SIRT1 as a molecular target. Furthermore, reviews were excluded from this study, selecting only original experimental studies.

Our search includes all the eligible articles in English regarding in vitro and in vivo animal studies.

### 2.3. Literature Search and Selection of Articles

**Figure 2:**
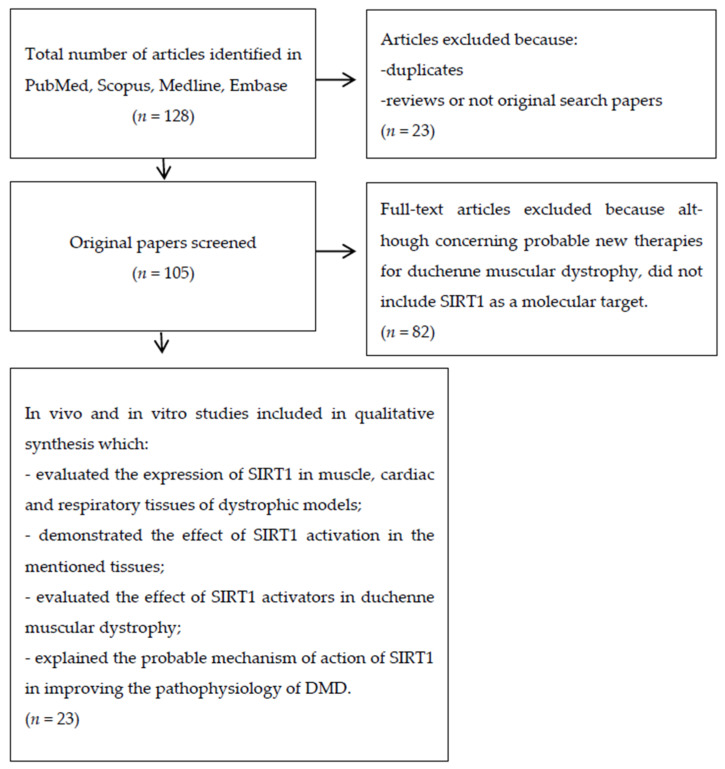
Prisma flow diagram represents the steps we followed during the literature search and article selection for this systematic review.

We searched PubMed, Embase and Cochrane databases using different keywords in order to identify all the studies concerning the hypothetical correlation between SIRT1 and DMD. The keywords used during the search were: “Sirtuin and Duchenne Muscular Dystrophy”, “Sirtuin and Muscular Dystrophy”, “SIRT1 and Muscular Dystrophy”, “SIRT1 and Duchenne Muscular Dystrophy”, “SIRT2 and Muscular Dystrophy”, “SIRT3 and Muscular Dystrophy”, “SIRT4 and Muscular Dystrophy”, “SIRT5 and Muscular Dystrophy”, “SIRT6 and Muscular Dystrophy”, “SIRT7 and Muscular Dystrophy”, “SIRT1 activators and Duchenne Muscular Dystrophy”, “Resveratrol and Duchenne Muscular Dystrophy”, “Resveratrol and Muscular Dystrophy”, “Quercetin and Duchenne Muscular Dystrophy”, “SIRT1 inhibitor and Duchenne Muscular Dystrophy” and “SIRT1 and utrophin”. Initially, all the papers were screened based on their title and the abstract. After this first selection phase, we read and analyzed the full text of the selected studies to verify that they satisfied the eligibility criteria. The PRISMA diagram below shows the steps followed during our literature search and evaluation (Figure 2).

### 2.4. Data Extraction

We identified one hundred and twenty-eight papers, of which twenty-three studies were included in this systematic review.

## 3. Results

### 3.1. Overview of Literature Search Results

As shown in Figure 2, after a first literature search, we identified a total of 128 articles. We excluded 23 articles and eliminated duplicates and other studies not eligible for different reasons, like reviews and/or articles not corresponding to the main argument (SIRT1 pathway and its implication in DMD). Finally, we systematically reviewed twenty-three studies.

### 3.2. Description of Articles Included in the Systematic Review

In total, we included twenty three articles for further analysis. Some articles reported both in vivo and in vitro studies, so we grouped the experiments according to the methodology used, respectively: twenty-two in vivo animal experimental studies, and seven in vitro experimental studies, among which five were in vitro animal studies and two were in vitro human studies.

#### 3.2.1. Main Outcomes Obtained by In Vivo Animal Studies

In Table 1, we summarize all the endpoints resulting from in vivo animal studies. In all the experiments, mdx mice, which have a mutation that leads muscle cells to produce a small, nonfunctional dystrophin protein, were used as an animal model of Duchenne muscular dystrophy. Fujiwara et al. observed pathological features similar to mice with muscular dystrophies in skeletal muscle-specific SIRT1 knockout mice (SIRT1-MKO) [37]. Moreover, Hulmi et al. highlighted an increase in phosphorylated SIRT1 (p-SIRT1), and a decrease in total SIRT1 in mdx mice when compared to wild type [38]. This evidence suggests the importance of SIRT1 activation in improving muscle function. Other studies have also shown the key role of SIRT1 activation in DMD disease; in particular, Chalkiadaki et al. elucidated how SIRT1 overexpression may influence the skeletal muscle properties of mdx mice. Initially, they generated SIRT1 transgenic mice, a mice model with higher SIRT1 muscle level, and then they observed that this modification resulted in a fiber shift from fast-to-slow twitch, which prevents muscular atrophy and dystrophy [39]. Moreover, they crossed SIRT1 muscle transgenic mice to mdx mice, and once again a notable improvement in the muscular parameters and performance was noted [39]. Hence, SIRT1 activation appears to be crucial for muscle fibers’ composition, mitochondrial genes and atrophy [39]. Different methods have been used in studies to increase SIRT1 expression or its activation, and to date, one of the most promising SIRT1 activators that can improve the clinical conditions in mdx mice seems to be resveratrol at a dose of 100 mg/kg/day [40,41]. Further studies are needed to affirm the effectiveness of SIRT1 activation as a new therapeutic target in DMD patients, and below we summarize the effect of the treatments in skeletal muscles, cardiac tissue and diaphragm.

##### Effects of SIRT1 Activation in Skeletal Muscle

A total of nine studies elucidated the role of SIRT1 activation in improving skeletal muscle properties and function of mdx mice. Two basic activators were used in the various experiments: resveratrol and quercetin, respectively, at different doses and for different periods of treatment.

Capogrosso et al. studied the effect of some natural compounds, such as resveratrol, taurine and apocynin, in ameliorating DMD pathophysiology, compared to α-methyl prednisolone (PDN), a corticosteroid largely used in this disease [8].The treatment lasted 4–5 weeks and the administration of these natural activators of SIRT1/PGC-1α pathway, in particular resveratrol at a dose of 100 mg/kg5 days/week, led to a reduction in superoxide anion production and to decreased plasma levels of creatine kinase and lactate dehydrogenase, hence muscle force is increased [8]. The study of Gordon et al. analyzed the effect of resveratrol in reducing muscle inflammation [41]. It was found that resveratrol, by increasing SIRT1 expression, decreased immune cells infiltration, reduced macrophage infiltration, enhanced PGC-1α activity, and increased utrophin levels (a dystrophin analog) [41]. These effects were observed for a short treatment period of only 10 days [41].

However, long-term treatment of resveratrol has also shown positive effects in improving the pathophysiology of DMD. In fact, it was found that daily oral intake or intraperitoneal injection (i.p.) for several weeks in mdx mice contributed to the preservation of muscle function and muscle mass [43,45,53]. These beneficial effects appear to be attributed to the activation of SIRT1 with consequent fiber shift from fast-to-slow twitch, which, as mentioned before, prevents muscular dystrophies [40,55].The optimal dose of resveratrol treatment in most of the experiments is shown to be 100 mg/kg/day [8,40,41,53,55].

Quercetin also proved to be a good activator of SIRT1 with beneficial effects against Duchenne muscular dystrophy. A long-term quercetin dietary intake prevents 50% loss of specific tension and fatigue resistance in skeletal muscle of mdx mice, as demonstrated by Spaulding et al. [51].

Another compound that exerts positive effects in skeletal muscle is adiponectin, a hormone with anti-inflammatory properties [35]. To prove its effectiveness in mdx mice, Abou-Samra et al. generated mdx-ApN mice by crossing mdx mice with mice overexpressing ApN [35]. mdx-ApN mice showed decreased muscle damage and enhanced muscle force compared to mdx mice; the effect seems to be due to SIRT1 activation, that in turn promotes the downregulation of inflammatory genes and the upregulation of utrophin [35].

##### Effects of SIRT1 Activation in Cardiac Tissue

Considering that cardiomyopathy is the main cause of death in DMD, different studies elucidated the role of SIRT1 activators in ameliorating cardiac function of dystrophic hearts. The SIRT1 activators used in these studies are, once again, resveratrol and quercetin.

Kuno et al. demonstrated that a long-term dietary intake of resveratrol for 32 weeks [42,44] or 56 weeks [46] inhibited hypertrophy and fibrosis in cardiac tissue of mdx mice, resulting in the maintenance of cardiac functions [42,44]. Overall, these data suggest that resveratrol improves mdx mouse survival.

Quercetin also appears to relieve cardiomyopathies in mdx mice. Ballman et al. proved that a 0.2% enriched quercetin diet administered for 6, 8 or 12 months prevented cardiac dysfunction of dystrophic hearts by decreasing the inflammatory markers and cardiac tissue damage, and by increasing mitochondrial biogenesis and utrophin expression [34,52,54].

##### Effects of SIRT1 Activation in Diaphragm

From the studies analyzed for the preparation of this systematic review, it emerged that the activation of SIRT1 has positive effects also in the diaphragm muscle, thus improving respiratory function, which generally fails in patients with DMD. A long-term intake of 0.2% enriched quercetin diet, administered for 6 months, lead to an increase in the number of muscle fibers and reduced fibrotic area, but fails to increase utrophin levels, suggesting that maybePGC-1α/SIRT1 pathway is only partially activated in diaphragm muscle [50]. In fact, Selsby et al. highlighted that a prolonged administration of quercetin for 12 months improved the respiratory function only for the first 6 months [47]. The insensitivity to the treatment seems to be a result of the decreased SIRT1 activity in mdx mice [47].

#### 3.2.2. Main Outcomes Obtained by In Vitro Studies

In Table 2, we report a schematic summary of the main endpoints resulting from in vitro studies. We identified five different studies conducted in animal cells where the experiments were performed in two different cell lines: myoblast cells and cardiomyocytes, respectively. We also analyzed two in vitro human studies, and in this case, the experiments were conducted in human myotubes.

##### SIRT1 Effects in C2C12 Myoblast Cells

Three studies examined the role of SIRT1 in myoblast cells; in particular, Fujiwara et al. observed that SIRT1 inhibition in C2C12 myoblast cells inhibits membrane resealing after injury [37]. In fact, they proved that cells treatment with a SIRT1 inhibitor, or Sirt1-silencing RNA (siRNA), alters the fusion of intracellular vesicles to the injured membranes, thus preventing their repairing mechanisms [37]. Furthermore, beneficial effects were observed from the activation of SIRT1 in myoblastic cells. Hori et al. demonstrated that the treatment of C2C12 cells with transforming growth factor-β1 (TGF-β1) led to increased reactive oxygen species (ROS) levels and fibronectin production [43]. However, resveratrol pretreatment of cells, followed byTGF-β1 treatment, reverses all these effects through SIRT1 activation, resulting in a containment of oxidative damage [43]. In C2C12 myoblast cells, resveratrol also promotes mitophagy processes of damaged mitochondria containing high superoxide species levels, resulting overall in reduced inflammation [45].

##### SIRT1 Effects in Cardiomyocytes

Two in vitro studies were performed in cardiomyocytes, since heart failure is one of the main causes of death in DMD patients. Progressive muscle degeneration leads to the replacement of muscle tissue with connective or adipose tissue, which loses its contractile function [4]. Kuno et al. analyzed how SIRT1 may exert its beneficial effect in cardiomyocytes. SIRT1 activation via resveratrol treatment promotes autophagy and mitophagy, leading overall to decreased ROS accumulation in heart [46], and hypertrophy inhibition through pro-hypertrophic co-activator p300 downregulation [44]. p300 protein is generally upregulated in mdx hearts and induces cardiomyocyte hypertrophy and tissue fibrosis [44]. Overall, these data confirm the efficacy of resveratrol in activating SIRT1, and consequently in relieving or slowing down the degradation process of cardiac muscle tissue in mdx mice. However, further studies are certainly needed before translating these results in DMD patients.

##### Main Outcomes Obtained by In Vitro Human Studies

In Table 2, we also report some evidence regarding the implication of SIRT1 pathway in human cells. Two experiments were performed in human myotubes. Cell cultures have been treated with adiponectin (ApN), a hormone with anti-inflammatory properties that has shown to have beneficial effects in mdx mice [35], and for this reason its activity has also been tested in human cells.

Lecompte et al. reported the effects of ApN following an inflammatory event in human myotubes of DMD and control patients [2]. Interestingly ApN activates the receptor AdipoR1, and consequently the AMPK-SIRT1-PGC-1α axis, leading to a cascade of biochemical events that ultimately result in the downregulation of two pro-inflammatory markers: tumor necrosis factor alpha (TNFα) and interleukin-17A (IL-17A); the upregulation of interleukin-6 (IL-6) with anti-inflammatory activity, and the upregulation of utrophin [2].

Samra et al. also elucidated the role of ApN in human myotube cells and showed that ApN treatment led to the downregulation of the nuclear factor kappa B (NF-κB), and the upregulation of utrophin [35]. To highlight the role of SIRT1 in this mechanism, they used silencing RNA encoding for AdipoR1, SIRT1 and PGC-1α and observed that the beneficial effects of ApN disappeared, thus clarifying the importance of the SIRT1-PGC-1α axis for the anti-inflammatory action of ApN [35].

Overall, these studies demonstrate the effectiveness of ApN, through AMPK-SIRT1-PGC-1α activation in reducing muscle inflammation.

## 4. Discussion

This systematic review includes 23 original papers, both animal (in vivo and in vitro), and in vitro human studies. The main aim of our systematic review is to assess the correlation between Duchenne muscular dystrophy and the enzyme SIRT1. We identified different studies and considered all the outcomes obtained. We found of relevant interest the information regarding the effects due to the lack of SIRT1 activity.

Fujiwara in his study found that SIRT1 knockout mice, characterized by suppressed SIRT1 expression, showed similar characteristics to dystrophic mice, such as reduced exercise capacity, muscle inflammation and muscle fragility [37]. In fact, in SIRT1 knockout mice high serum levels of some muscle injury markers such as CK and LDH, necrotic fibers and inflammatory changes were observed. Altered parameters are shown in C2C12 cell cultures of myoblasts too, where reduced SIRT1 activity led to a failure in repairing damaged cells membranes after injury, due to muscular membrane fragility caused by genetic mutations of the dystroglycan complex [37]. Conversely, by activating the PGC-1α/SIRT1 pathway, several beneficial effects have been observed. In fact, SIRT1 overexpression in mdx muscles leads to a fiber shift from fast to slow twitch, and fibers with a smaller size, which results in an increase in oxidative fibers [39]. The expression levels of different fibers type markers were evaluated, such as troponin slow, a marker of oxidative fibers, which is increased by SIRT1 overexpression. SIRT1 overexpression also affects the expression of myosin heavy chain isoforms MHC-I and MHC-2 (markers of oxidative fibers too) that seem to be increased, while the expression of MHC-2B isoform (marker of fast-twitch fibers) is reduced [39]. These findings are correlated with an increased expression of PGC-1α, which in turn is positively activated by SIRT1 deacetylation. PGC-1α overexpression or activation promotes mitochondrial biogenesis and oxidative fibers as well. Overall, these conditions reverse the characteristics of dystrophic muscles, ameliorating the disease and improving muscle function [39].

The active form of SIRT1 is the phosphorylated one [38] and its activation is determined by various circumstances. For this reason, we reviewed different papers that showed the effect of some exogenous agents that can enhance the expression/activity of SIRT1, improving DMD pathology. Resveratrol, a natural compound with antioxidant properties [40], is the main activator used in various studies and its excellent efficacy has been observed in the skeletal muscles, in the cardiac tissue, and at a respiratory level as well.

In skeletal muscles, the treatment of mdx mice with resveratrol leads to a reduction in superoxide anion production, creatine kinase plasma level and lactate dehydrogenase plasma level [8,45], all parameters of muscle inflammation. Its long-term dietary intake helps in the reduction in myofiber wasting and in the enhancement of muscular maturation [45]. Muscle mass and its composition are also improved: myofiber loss is reduced [43], myofibroblast cells are reduced [43] and the expression of slow, oxidative fibers is promoted [40]. Additionally, resveratrol implementation of dystrophic animal models drastically improves muscle inflammation: immune cell infiltration was reduced, macrophage infiltration was decreased, the expression of anti-inflammatory markers such as the IL-6 gene was increased, and ROS levels were decreased, all compared to untreated dystrophic mice [41,43].

Resveratrol treatment also promotes the PGC-1α signaling enhancing phenotype in mdx mice and improves utrophin gene expression [41]. The effect of resveratrol is dose-dependent and the most effective one seems to be 100 mg/kg/day [40,41].

Since the main cause of death in patients with DMD is cardiomyopathy, we also assessed the studies that demonstrated the efficacy of activating SIRT1 in heart. Resveratrol treatment of dystrophic mice also improves the cardiomyopathy of dystrophic hearts. In fact, resveratrol treatment suppressed cardiac hypertrophy of dystrophic mice: the heart weight of treated mdx mice is decreased compared to untreated mdx mice; the heart weight/body weight of untreated mdx mice is increased compared to control, but resveratrol reverses the increase [44]. Resveratrol treatment preserves also the cardiac function demonstrated by the downregulation of p300 protein levels in treated mdx mice [44] and by the increase in the mitophagy process that promotes the damaged mitochondrial deletion [46].

Similar evidence was observed in cultured cells of C2C12 myoblast cells, where resveratrol treatment decreased ROS levels and fibronectin synthesis [33], and promoted mitophagy [51]; and in cardiomyocytes where it downregulated p300 activity and promoted FoxO3a activity in the nucleus of cells leading overall to a decreased ROS accumulation in heart [46].

However, interesting results were also observed with quercetin, another natural SIRT1 activator. In quercetin-treated mdx mice, physical activity is increased compared to the untreated ones and they show a better muscle profile with an increase in muscle fibers, a reduction in fibers with centralized nuclei, an increased muscle mass, a reduction in immune cell infiltration, and a decrease in fibrotic area [51]. Muscle function is also affected by quercetin treatment: loss of specific tension and fatigue resistance were prevented [51].

Furthermore, quercetin treatment results in a reduction in the pathological remodeling of dystrophic hearts. The heart weight of untreated dystrophic mice is increased compared to control, but quercetin lowers the value [52]. The dietary intake of quercetin also lead to a reduction in the amount of fibronectin in mdx mice compared to the untreated ones [34], to a decrease in inflammatory marker levels, and to an increase in the protein level of PGC-1α, suggesting an improved mitochondrial biogenesis and reduced cardiac inflammation and damage [34,52,54].The positive effects of quercetin were observed also in ameliorating respiratory function by increasing muscle fibers, reducing fibrotic area and decreasing immune cell infiltration in diaphragm [50]. However, these effects seemed to be transient, so further examinations are needed [47]. In fact, respiratory frequency was increased compared to untreated mdx mice and was similar to control mice for the first 6–8 months of age, but beyond the 8th month there was not any improvement. Other parameters of respiratory function are also affected by quercetin treatment, such as tidal volume, minute ventilation, peak inspiratory flow, and peak expiratory flow, which are increased compared to untreated mdx mice [47].

The beneficial effects deriving from the activation of SIRT1 in animal models of DMD prompted the authors to study the effects that this activation may have on human cells too. We collected two studies performed on human myotubes and the cell cultures were treated with adiponectin, a hormone with anti-inflammatory properties [35]. In dystrophic myotubes ApN levels are decreased and its complementation leads to a downregulation of the pro-inflammatory marker TNFα, to an upregulation of the anti-inflammatory IL-6, and to an upregulation of utrophin A, a dystrophin analogue [2,35]. These positive effects of ApN are abrogated by siRNA silencing of genes encoding for AdipoR1, SIRT1, or PGC-1α suggesting that SIRT1/PGC-1α axis plays a crucial role in the anti-inflammatory activity of ApN [35].

Although further research is needed to clarify the molecular mechanisms underlying the protective role of SIRT1 in DMD, we propose SIRT1 as a novel hypothetical therapeutic target for patients with muscular dystrophies.

## 5. Conclusions

Muscular dystrophies are hereditary muscular disorders characterized by a progressive loss of muscle composition and function. Duchenne muscular dystrophy is one of the most common and severe forms, and is caused by a mutation in the gene encoding for dystrophin protein. Dystrophin deficiency leads to an alteration of the dystrophin glycoprotein complex resulting in a multitude of molecular alteration that ultimately lead to muscle degeneration and myofiber necrosis [1,2,3]. To date, the only effective therapy is represented by glucocorticoids administrations, which on the other hand are associated with multiple side effects. Therefore, the identification of new pharmacological targets represents an excellent prospect of current interest.

SIRT1, a NAD+-dependent histone/protein deacetylase, aroused considerable interest in us due its role in different cellular processes, including stress resistance, inflammatory processes and cell survival. SIRT1 is expressed in different tissues, and we focus on its role in skeletal muscle since experimental studies have shown that SIRT1 activation provides beneficial effects in the dystrophin-deficient mdx mouse. SIRT1 promotes the deacetylation, and consequently the activation of PGC-1α, which in its active form controls mitochondrial biogenesis and homeostasis through mitophagy and autophagy processes, improves muscle resistance, enhances muscle fiber-type switching, and decreases the process of muscle wasting due to its ROS scavenging activity and due to its role in promoting pro-inflammatory markers level.

However, the main causes of death of DMD patients are cardiac and respiratory arrest, and we found the role of SIRT1 also interesting in cardiac and respiratory muscle tissue. At the cardiac level, the activation of the SIRT1-PGC-1α axis inhibits cardiac hypertrophy, characteristic of dystrophic models, and improves cardiac function.

Respiratory function is also enhanced by SIRT1 activation, but in this case, the effect is transient, so further studies are needed to elucidate its role in respiratory muscles.

In fact, from the experimental studies collected in the literature, the techniques used to implement SIRT1 activity were the generation of transgenic mice which show SIRT1 overexpression, or the use of specific SIRT1 activators such as resveratrol (especially effective at the dose of 100 mg/kg/day) and quercetin.

The beneficial effects deriving from SIRT1 activation have been found in human cells too. In this case, adiponectin, a hormone with anti-inflammatory properties, was performed to increase SIRT1 activity, leading to downregulation of the pro-inflammatory marker TNFα, to an upregulation of the anti-inflammatory IL-6 and to an upregulation of utrophin A, a dystrophin analogue [35]. These findings lead the research to move further by using SIRT1 as a molecular target in DMD.

However, all these findings taken together are only the beginning of a major study necessary to prove the real efficacy of SIRT1 as a new pharmacological target in the prevention of muscle degeneration in DMD patients, but they can still be considered a good starting point.

## Figures and Tables

**Table 1:** Overview of the characteristics of in vivo animal studies included in the systematic review.

No	Study	Type of Animal	Experimental Design	Event	Parameters Assessed	Outcomes
1	Hulmi, J.J. et al. “Effects of muscular dystrophy, exercise and blocking activin receptor IIB ligands on the unfolded protein response and oxidative stress.” Free Radical Biology and Medicine 99 (2016) 308–322	mdx mice/wild type mice as control	(1) Comparison between mdx and wild type mice regarding: endoplasmatic reticulum (ER) stress and unfolded protein response (UPR); the ratio of phosphorylated-SIRT1 (p-SIRT1) and SIRT1; acetylated lysine levels. (2) Seven weeks of voluntary exercise and/or soluble activin receptor-Fc (sAcvR2B-Fc) administration.	Dystrophin deficiency in skeletal muscle.	ER stress/UPR; Phosphorylated SIRT1 levels; Acetylated protein lysine residues levels.	(1) Compared to wild type mice, in mdx mice UPR response is activated in the ER, also p-SIRT1 levels are increased 1.7 times and total SIRT1 levels decreased leading to increased p-SIRT1/SIRT1. Further more acetylated protein lysine residues are increased 1.6 times in mdx mice compared to the wild type ones. All these conditions contribute to the onset of DMD pathophysiology. (2) Voluntary exercise alone or combined with sAcvR2B-Fc administration leads to increased p-SIRT1 which in turn lead to redox regulation [38].
2	Capogrosso, R.F. et al. “Assessment of resveratrol, apocynin and taurine on mechanical-metabolic uncoupling and oxidative stress in a mouse model of duchenne muscular dystrophy: A comparison with the gold Standard, α -methyl prednisolone.” Pharmacological Research 106 (2016) 101–113	mdx mice/wild type mice as control	Resveratrol (100 mg/kg i.p 5 days/week); apocinin (38 mg/kg/day per os); taurine (1 g/kg/day per os); α-methyl prednisolone (1 mg/kg i.p., 5 days/week) were administered for 4 weeks in parallel with twice a week exercise (30 min running on a horizontal treadmill).	Evaluation of resveratrol, apocinin and taurine in comparison with methyl prednisolone (PDN) in ameliorating DMD.	Body weight; fore-limb force; plasma levels of creatine kinase and lactate dehydrogenase; ROS levels; NADPH oxidase (NOX) activity; SIRT1 expression.	(1) Body weight: In wt untreated mice, the increment of body weight was lower (+7 g) compared to untreated mdx mice (+8.5/+11.8 g). mdx mice treated with apocynine or taurine showed no great differences compared to the untreated ones. Conversely resveratrol (+7 g), and especially PDN (+3 g) lowered dhe body weight increment. (2) Forelimb force: similar values for untreated wt and mdx mice; increased values for treated mdx mice with increased recovery score (RS): apocynin 35%; taurine 55%; PDN 58%; resveratrol 60%. (3) Creatine kinase: resveratrol reduced CK levels with a rescue score of 64%; taurine, apocynine and PDN did not affect CK levels. (4) Lactate dehydrogenase: resveratrol and taruine reduced LDH with a rescue score of 82% and 47%, respectively; apocynine and PDN did not affect LDH levels. (5) SIRT1 expression: all the treatments led to an unexpected reduction in SIRT1 expression, probably due to an ROS reduction after the treatment [8].
3	Kuno, A. et al. “The effects of resveratrol and SIRT1 activation on dystrophic cardiomyopathy”. Ann. N.Y. Ann. N. Y. Acad. Sci. 1348 (2015) 46–54	mdx mice/wild type mice as control.	Resveratrol (4 g/kg chow ad libitum) for 32 weeks, beginning at the age of 9 weeks. Estimated intake of resveratrol: 500 mg/kg/day.	Resveratrol’s effect on cardiomyopathy due to muscular dystrophy.	Motion of the left ventricular wall; cardiac hypertrophy; myocardial mRNA levels of B-type natriuretic peptide; average life span; cardiomyocyte cross-sectional area; transcription co-activator p300.	Resveratrol treatment suppressed cardiac hypertrophy due to a suppressed increase in heart weight body weight ratio, reduced myocardial mRNA levels of atrial natriuretic peptide and cardiomyocites cross-sectional area, compared to untreated mice. It also preserved cardiac function and reduced tissue fibrosis in diseased heart [42].
4	Chalkiadaki, A. et al. “Muscle-Specific SIRT1 Gain-of-Function Increases Slow Twitch Fibers and Ameliorates Pathophysiology in aMouse Model of Duchenne Muscular Dystrophy”. PLoS Genetics (2014)	Muscle-specific transgenic mice (TG)/SIRT1 muscle-specific knockout mice (MckKO).	SIRT1 muscle transgenic mice were crossed to mdx mice.	Effect of SIRT1 overexpression in muscles.	Muscle wasting; alteration of fiber composition; PGC-1α activity; utrophin levels.	Mice with overexpressed SIRT1 (TG mice) oppose atrophy gene program reversing muscle hypertrophy of dystrophic muscle, improved fiber shift from fast-to-slow twitch, increased utrophin levels and increased PGC-1α levels compared to MckKO [39].
5	Fujiwara, D. et al. “SIRT1 deficiency interferes with membrane resealing after cell membrane injury”. PLoS ONE 14(6) (2019).	Skeletal muscle-specific SIRT1 knockout (SIRT1-MKO)/wild type mice as control.	Skeletal muscle-specific SIRT1 knockout mice (SIRT1 MKO) were generated.	Effect of lack of SIRT1 in skeletal muscles.	Exercise capacity; myofiber damage evaluation; membrane repair; serum levels of creatine kinase (CK) and lactate dehydrogenase activities (LDH).	SIRT1-MKO mice shows a similar pathophysiology to mild dystrophic mice. In SIRT1-MKO mice, high serum CK and LDH levels were observed, compared to the WT ones. Increased numbers of centrally nucleated small myofibers and decreased numbers of middle-sized myofibers were also observed in SIRT1-MKO mice compared to WT mice [37].
6	Gordon, B.S. et al. “Resveratrol decreases inflammation and increases utrophin gene expression in the mdx mouse model of duchenne muscular dystrophy”. Clinical Nutrition 32 (2013) 104–111.	mdx mice.	Resveratrol (0, 10, 100, or 500 mg/kg) everyday for 10 days beginning at the age of 5 weeks.	Effect of resveratrol in reducing inflammation caused by DMD.	SIRT1 gene expression; active dose of resveratrol; immune cell infiltration; macrophage infiltration; IL-6, PGC-1α, and utrophin levels.	Resveratrol treatment improved SIRT1 gene expression, especially at the dose of 100 mg/kg where the increase is about 60 ± 10%. So, the most effective dose (100 mg/kg) was used for further analysis: - Immune cell infiltration was reduced by about 42 ± 8%. - Macrophage infiltration was decreased by 48 ± 10%. - IL-6 gene expression was increased by about 247 ± 77%. - PGC-1α gene expression was increased by about 27 ± 17%. - TNFα gene expression was unchanged. - Utrophin gene expression was increased by about 43 ± 23%. Overall inflammation is significantly reduced after resveratrol treatment [41].
7	Hori, Y.S. et al. “Resveratrol Ameliorates Muscular Pathology in the Dystrophic mdx Mouse, a Model for Duchenne Muscular Dystrophy” The Journal of pharmacology and experimental therapeutics (2011) 338:784–794	mdx mice/wild type mice as control	Resveratrol (4 g/kg meal) mixed with powder meal and orally administered ad libitum for 32 weeks, beginning at the age of 9 weeks.	Resveratrol’s effect in mdx mice.	Preservation of muscle mass; inhibition of oxidative stress and fibrosis.	After resveratrol treatment, the following parameters were assessed, compared to mdx untreated mice: - Myofiber loss was reduced by about 20%; - ROS levels were decreased by about 50%; - Myofibroblast cells were reduced by about 75%. Inflammatory cells’ infiltration and TGF-β1 citokine release were not affected by resveratrol treatment [43].
8	Ljubicic, V. et al. “Resveratrol induces expression of the slow, oxidative phenotype in mdx mouse muscle together with enhanced activity of the SIRT1-PGC-1 axis”. Am J Physiol Cell Physiol (2014) 307: C66–C82.	mdx mice/wild type mice as control	-First protocol: resveratrol (100 mg/kg/day with diet) for 6 weeks. Moderate dose and short duration (MDSD). -Second protocol: resveratrol (500 mg/kg/day with diet) for 12 weeks. High dose and long duration (HDLD).	Effect of resveratrol in muscle remodeling by stimulating the slow, oxidative myogenic program in dystrophic skeletal muscle.	SIRT-1 activity; PGC-1α activity.	Resveratrol treatment promotes SIRT1 and PGC-1α signaling, enhancing the expression of the slow, oxidative phenotype in mdx mice. The dose of 100 mg/kg/day maximizes the effect [40].
9	Kuno, A. et al. “Resveratrol Improves Cardiomyopathy in Dystrophin-deficient Mice through SIRT1 Protein-mediated Modulation of p300 Protein”. The Journal of Biological chemistry, vol. 288 (2013) 5963–5972	mdx mice/control mice	Resveratrol (4 g/kg) mixed with powder meal and orally administered ad libitum for 32 weeks, beginning at the age of 9 weeks.	Effect of SIRT1 modulation, through long-term resveratrol treatment, in cardiomyopathy.	p300levels; SIRT1 activity; heart weight.	Resveratrol treatment ameliorated cardiomyopathy and cardiac function in mdx mice. Below are some of the parameters assessed that proved it: - Heart weight of mdx mice is increased compared to control (from ±150 mg to ±200 mg); resveratrol treatment lowers the value from ±200 mg to ±150 mg, compared to untreated mdx mice; - Heart weight/body weight of untreated mdx mice is increased from ±4 to ±6 mg/g compared to control, but resveratrol reverses the increase; - p300 levels are increased 2.5 times in untreated mdx mice compared to control mice; resveratrol treatment reverses this enhancment. Overall hypertrophy and fibrosis are inhibited by resveratrol treatment [44].
10	Sebori, R. et al. “Resveratrol Decreases Oxidative Stress by Restoring Mitophagyand Improves the Pathophysiology of Dystrophin-Deficientmdx Mice”. Oxidative Medicine and Cellular Longevity Volume (2018)	mdx mice/control mice	Resveratrol (0.04, 0.4, and 4 g/kg food) for 56 weeks.	Resveratrol effect in dystrophin-deficient mdx mice through oxidative stress reduction.	Creatine kinase levels; physical activities; autophagy.	In treated mdx mice reduced myofiber wasting and enhanced muscular maturation were observed at the dose of 4 g/kg food of resveratrol compared to untreated mdx mice. Additionally, reduced creatine kinase levels and increased physical activity at the dose of 0.4 g/kg food of resveratrol were shown, compared to untreated mdx mice [45].
11	Kuno, A. et al. “Resveratrol Ameliorates Mitophagy Disturbance and Improves Cardiac Pathophysiology of Dystrophin deficient mdx Mice”. Scientific reports (2018) 8:15555	mdx mice/ control mice	Resveratrol (0.04, 0.4, or 4 g/kg food) ad libitum, mixed with powdered meal for 56 weeks, beginning at the age of 9 weeks.	Benefits of resveratrol in cardiac diseases in mdx mice, by promoting mitophagy.	Mitochondrial DNA (mtDNA); autophagy of damaged mitochondria (mitophagy) level; ROS levels.	Mdx mice shows lower levels of cardiac mtDNA compared to control mice. Resveratrol treatment improved cardiomyopathy, increasing mitophagy and decreasing mtDNA deletion and ROS levels compared to untreated mdx mice. The most effective dose is 0.4 g/kg food of resveratrol [46].
12	Ballmann, C. et al. “Lifelong quercetin enrichment and cardioprotection in Mdx/Utrn/mice”. Am J Physiol Heart CircPhysiol (2017) 312: H128–H140.	dystrophin-deficient mice heterozygous for a utrophin mutation (Mdx/Utrn^+^/^−^)/ control mice	0.2% quercetin enriched diet, provided ad libitum for 8 months, beginning at the age of 2 months.	Cardioprotective effect in dystrophic hearts of quercetin, a SIRT1/PGC-1α activator.	Functional cardiac assessment; cardiac expression of utrophin, fibronectin, inflammatory markers.	Quercetin treatment results in a reduction in the pathological remodeling of the dystrophic heart; below are listed some of the parameters assessed: - Body weight of treated and untreated Mdx/Utrn^+^/^−^ is similar (42.0 ± 2.34 g and 41.2 ± 2.73 g, respectively) and greater then control mice (30.8 ± 2.50 g). - Heart weight of untreated Mdx/Utrn^+^/^−^ is increased compared to control mice (194 ± 15.31 mg and 162 ± 11.45 mg, respectively); quercetin treatment lowers the value to 183 ± 13.6 mg in Mdx/Utrn^+^/^−^ mice. - mitochondrial protein markers of oxidative stress and endogenous antioxidant enzyme are also improved after quercetin treatment, resulting in decreased cardiac damage tissue [34].
13	Selsby, J.T. et al. “Oral quercetin administration transiently protects respiratory function in dystrophin-deficient mice”. J Physiol 594.20 (2016) pp 6037–6053	mdx mice/control mice	0.2% quercetin enriched diet, provided ad libitum for 12 months, beginning at the age of 2 months.	The benefits of dietary quercetin supplementation in respiratory function of dystrophin-deficient mice.	Respiratory function; histological parameters; biochemical parameters.	Quercetin treatment improved respiratory function; in fact respiratory frequency was increased compared to untreated mdx mice and was similar to control mice for the first 6–8 months of age, but beyond the 8th month there is not any improvement. The maximum value was obtained at 8 months of age where the frequency of treated mdx mice was ±115, versus ±95 for untreated mdx mice. Quercetin treatment also improved other parameters of respiratory function such as tidal volume, with a 20-fold increase, minute ventilation, with a 50-fold increase, peak inspiratory flow, with a 25-fold increase, and peak expiratory flow, with a 40-fold increase, compared to untreated mdx mice [47].
14	Abou-Samra, M. et al. “Involvement of adiponectin in the pathogenesis of dystrophinopathy”. Skeletal Muscle (2015) 5:25.	mdx-ApN mice/mdx mice/wild type mice as control	Mice overexpressing adiponectin (ApN mice) are crossed with mdx mice in order to obtain mdx mice overexpressing ApN (mdx-ApN mice).	Effect of adiponectin in counteracting muscle degeneration.	Global force or resistance; muscle damage markers in plasma; plasma creatine kinase (CK) and lactate dehydrogenase (LDH); utrophin levels.	Different parameters were assessed in the three group of animals: - Body weight was similar, with a value 22–23 g; - Tibialis anterior muscle pair weight was increased in mdx mice compared to control mice (92 ± 6 mg and 72 ± 2 mg, respectively); the overexpression of adiponectin in mdx-ApN mice lowered the value to 76 ± 5 mg; - PlasmaApN levels in mdx mice were lower compared to control mice (2.6 ± 0.2 μg/mL and 4.2 ± 0.2 μg/mL, respectively, while mdx-ApN mice had similar levels like controls 3.6 ± 0.1μg/mL. The increased ApN levels in mdx-ApN mice was accompanied by a reduction in muscle inflammation and oxidative stress and an increase in muscle function due to utrophin upregulation (a dystrophin analog) [35].
15	Camerino, G., M. et al. “Gene expression in mdx mouse muscle in relation to age and exercise: aberrant mechanical–metabolic coupling and implications for pre-clinical studies in Duchenne muscular dystrophy”. Human Molecular Genetics, (2014) 1–13.	mdx mice/wild type mice as control	Twice a week, mdx and control mice undergo to 30 min running on a horizontal treadmill at 12 m/min, with a break of 48–72 h between each session. The exercise lasts for 4 or 12 weeks.	Age and exercise -related gene expression in mdx mice muscles.	Effect of exercise on in vivo performance; analysis of gene expression in the GC muscles of mdx.	The effect of exercise on in vivo performance were demonstrated with parameters such as: - Forelimb strength: exercise did not affect absolute and normalized forelimb strength of wild type mice, but it caused a reduction in both the values in mdx mice starting from 4 weeks of exercise and it was maintained until 12 weeks of exercise. - PGC-1α gene expression: after 4 weeks, exercise increased PGC-1α expression in GC muscles of wild type mice; in non-exercised mdx mice, there was a basal upregulation of PGC-1αcompared to wild type mice. Conversely, in mdx mice, 4 weeks of exercise did not affect PGC-1α gene expression. - SIRT1 gene expression: did not change after 4 or 12 weeks of exercise in wild type mice. However, it was upregulated in sedentary mdx mice versus WT at both ages [48].
16	Ljubicic, V. et al. “Chronic AMPK stimulation attenuates adaptive signaling in dystrophic skeletal muscle”. Am J Physiol Cell Physiol (2012) 302: C110–C121	mdx mice	AMP-activated protein kinase (AMPK) activator 5-aminoimidazole-4-carboxamide-1—D-ribofuranoside (AICAR; 500 mg/kg/day) for 30 days, and then half of the animals were subjected to treadmill running to induce acute AMPK signaling.	Effects of induced phenotype shift in dystrophic skeletal muscle in the subsequent intracellular signaling.	Evaluation of mRNA levels of phenotypic modifiers, including PPARγ coactivator-1α (PGC-1α) and SIRT1.	In mdx mice, treadmill running induces PGC-1α, and SIRT1 mRNAs, that in turn promotes the slow, oxidative myogenic program. Additionally, acute stress-induced expression of PGC-1α and SIRT1 is attenuated by AMPK stimulation. These data suggest the importance of the axis PPGC-1α/SIRT1 as a novel therapeutic target for DMD [49].
17	Hollinger, K. et al. “Long-term quercetin dietary enrichment decreases muscle injury in mdx mice”. Clinical Nutrition (2015) 34(3):515–522	mdx mice	0.2% quercetin enriched diet, provided ad libitum, for 6 months, beginning at the age of 3 months.	Effect of long-term quercetin enriched diet in decreasing muscle injury in diaphragm of dystrophic mice.	In a diaphragm strip: number of muscle fibers, percentage of fibers with centralized nuclei, fibrotic area, and expression of oxidative genes.	Quercetin-treated mdx mice show a better muscle profile with an increase of 24% in muscle fibers, a reduction of 34% in fibers with centralized nuclei; a 31% reduction in immune cell infiltration and a 47% decrease in fibrotic area, all compared to untreated mdx mice. Utrophin levels are unchanged [50].
18	Spaulding, H., R. et al. “Long-Term Quercetin Dietary Enrichment Partially Protects Dystrophic Skeletal Muscle” PLoS ONE (2016) 11(12)	mdx mice/wild type mice as control	0.2% quercetin-enriched diet, provided ad libitum, for 12 months, beginning at the age of 2 months.	Effect of long-term quercetin-enriched diet in preservation of limb muscle function through PGC-1α/SIRT1 pathway activation.	Physical activity; soleus and extensor digitorumlongus (EDL) muscle function; specific tension; fatigue resistance; muscle injury; fibrosis.	In quercetin-treated mdx mice, physical activity is increased. In total, 50% of loss of specific tension and fatigue resistance in the soleus is prevented, while in EDL muscle there are no big improvements. Relative muscle mass in the soleus is doubled in untreated mdx mice compared to control, and in the treated one it is 11% greater than in the untreated ones. In EDL muscle, the relative muscle mass of untreated mdx mice is 60% increased compared to control mice, but no improvements are observed after quercetin treatment [51].
19	Ballmann, C. et al. “Long term dietary quercetin enrichment as a cardioprotective countermeasure in mdx mice”. Exp Physiol. (2017) 102(6):635–649.	mdx mice/control mice	0.2% quercetin enriched diet, provided ad libitum, for 12 months, beginning at the age of 2 months.	Cardioprotective effect of long-term quercetin enriched diet in mdx mice.	Physical activity; cardiac function; percentage of damaged tissue; cardiac levels of fibronectin.	Different parameters were assesed: - Body weight and heart weight were similar for all groups of animals; - Cardiac function in untreated mdx mice is decreased in a time dependent-manner, and unexpectedly, quercetin treatment does not lead to big improvements. - Fibronectin levels in quercetin-fed mdx mice are reduced compared to untreated mdx mice. Inflammatory markers levels are decreased and the protein abundance of PGC-1α is increased, suggesting an improved mitochondrial biogenesis and reduced cardiac inflammation [52].
20	Gordon, B.S. et al. “Resveratrol improves muscle function but not oxidative capacity in young mdx mice”. Can. J. Physiol. Pharmacol. Vol. 92, (2014)	mdx mice	Resveratrol (100 mg/kg suspended in 200 µL of water every other day) for 8 weeks beginning at the age of 4–5 weeks.	Resveratrol effects on muscle function, muscle pathology and oxidative capacity in young mdx mice.	Body grip strength; in situ muscle function; fibers total number; centralized nuclei cells; number of immune cells; utrophin quantification.	Different parameters were assessed to identify the role of resveratrol treatment in muscle function: - Body grip strength: no improvements are shown between treated and untreated mdx mice; - Rotarod performance: in mdx treated mice there is an increase of 53 ± 20% compared to untreated mdx mice; - Specific peak tension of the triceps: after resveratrol treatment is increased about 17 ± 4% compared to untreated animals; - Number of central nuclei cells: is decreased by 12 ± 4% after resveratrol treatment; - Immune cell infiltration: is not affected by the treatment; - Expression of pro-inflammatory genes: is not affected by resveratrol treatment [53].
21	Ballmann, C. et al. “Histological and Biochemical Outcomes of Cardiac Pathology in mdx Mice with Dietary Quercetin Enrichment“. ExpPhysiol 100.1 (2015) 12–22	Mice	Experiment 1: 0.2% quercetin enriched diet, ad libitum, for 6 months, beginning at the age of 3 weeks. Experiment 2: 0.2% quercetin enriched diet, ad libitum, for 6 months, beginning at the age of 3 months.	Effect of quercetin-enriched diet in preventing and rescuing cardiac pathology in mdx mice.	Mitochondrial biogenesis; cardiac remodeling; fibrotic area; antioxidant expression; utrophin levels.	Protocol 1: Prevention. Quercetin increases mitochondrial biogenesis in hearts and improves heart levels of SOD2, an endogenous antioxidant enzyme. Utrophin levels are also increased. Protocol 2: Rescue. Quercetin feeding leads to a reduction in heart remodeling. Additionally, TGF-β1 levels, a regulator of fibrosis, are decreased. All these findings suggest that oral quercetin may be a counter measure against cardiac and skeletal muscle pathology [54].
22	Selsby, J.T. et al. “Rescue of Dystrophic Skeletal Muscle by PGC-1a Involves a Fast to Slow Fiber Type Shift in the mdx Mouse” PLoS ONE 7(1) (2012).	mdx mice	Overexpression induction of PGC-1α: (a) via injection of adeno-associated virus (AAV) (b) orally by administration of resveratrol (100 mg/kg/day) for eight weeks.	Effect of PGC-1α overexpression in rescuing dystrophic skeletal muscle.	Utrophin expression; type I myosin heavy chain expression; mitochondrial protein expression; SIRT-1 levels; muscle resistance; fatigue resistance.	Both treatments lead to an increased expression of PGC-1α, that in turn leads to increased utrophin levels, and type I myosin heavy chain as well. Mitochondrial protein expression and SIRT1 levels are also increased, while p38 and NRF-1 activity is reduced. All these events result in improved muscle and fatigue resistance [55].

**Table 2:** Overview of the characteristics of in vitro animal and human studies included in the systematic review.

No	Study	Type of Cells	Experimental Design	Event	Parameters Assessed	Outcomes
1	Fujiwara, D. et al. “SIRT1 deficiency interferes with membrane resealing after cell membrane injury”. PLoS ONE 14(6) (2019).	C2C12 myoblast cells	Cells treatment with vehicle or SIRT1 inhibitors: nicotinammide (NAM) 10 mM or Ex527 10 μM, incubated for 12 h. RNAi-mediated knockdown was performed by transfection of Sirt1-siRNAor Control-siRNA, both 30 nM.	Effects of SIRT1 inhibition in membrane resealing of C2C12 myoblast cells.	Effects of SIRT1 on membrane repair.	Influx of fluorescent dye FM1-43 in cells after laser irradiation was used to monitor membrane resealing. In treated C2C12 cells, FM1-43 uptake is improved compared to untreated cells, suggesting that NAM and Ex527 inhibited membrane resealing through SIRT1 inhibition.Treatment of cells with Sirt1-siRNA was used to further confirm the role of SIRT1 in membrane resealing. Additionally, in this case, in treated cells, membrane resealing is inhibited due to inhibited aggregation of membrane vesicles at the injured site [37].
2	Hori, Y.,S. et al. “Resveratrol Ameliorates Muscular Pathology in the Dystrophic mdx Mouse, a Model for Duchenne Muscular Dystrophy”. J Pharmacol. Exp. Ther. (2011) 338(3)784–94	C2C12 myoblast cells	Pre-treatement with resveratrol (30 µM) and then treatment with TGF-β1 (10 ng/mL)	Effects of resveratrol in reducing ROS levels in myoblasts, through SIRT1 activation.	Intracellular ROS levels.	TGF-β1 treatment leads to increased ROS levels and fibronectin synthesis, suggesting oxidative damage. Resveratrol pre-treatment reverses all these effects through SIRT1 activation [43].
3	Kuno, A. et al. “Resveratrol Improves Cardiomyopathy in Dystrophin-deficient Mice through SIRT1 Protein-mediated Modulation of p300 Protein”. THE JOURNAL OF BIOLOGICAL CHEMISTRY (2013). 5963–5972.	Cardiomyocytes of mdx mice.	Resveratrol (4 g/kg meal) mixed with powdered meal, orally administered ad libitum, for 32 weeks, beginning at the age of 9 weeks.	Downregulation of p300 as a cardioprotective mechanism of resveratrol, through SIRT1 activation, in DMD.	Activity of p300 protein; mechanism of p300 downregulation.	In untreated mdx mice the minimal Feret’s diameter of cardiomyocytes in the left ventricle was larger compared to those in control mice. Resveratrol treatment reverses the effect. Additionally, resveratrol and SIRT1 overexpression downregulate p300 activity in cardiomyocytes resulting in hypertrophy inhibition [44].
4	Sebori, R. et al. “Resveratrol Decreases Oxidative Stress by Restoring Mitophagy and Improves the Pathophysiology of Dystrophin-Deficient mdx Mice”. Oxidative Medicine and Cellular Longevity (2018).	C2C12 myoblast cells	Cells incubation with vehicle or resveratrol 30 µM for 6 h.	Effects of resveratrol in autophagy/mitophagy processes in C2C12 myoblast cells.	Mitochondrial superoxide levels in C2C12 cells; mitophagy; autophagy.	Resveratrol treatment of C2C12 cells improves autophagosome production and autophagy [56,57]. To assess the role of resveratrol in ROS reduction, antimycin (AA), a mitochondria depolarizer, was used. AA increases mitochondrial ROS levels in C2C12 cells, while resveratrol treatment suppressed this effect. Additionally, resveratrol improves mitophagy and reduces damaged mitochondria from C2C12 cells [45].
5	Kuno, A. et al. “Resveratrol Ameliorates Mitophagy Disturbance and Improves Cardiac Pathophysiology of Dystrophindeficient mdx Mice”. SCientifiCREPOrTS (2018) 8:15555	H9C2 cardiomyocytes	Vehicle or resveratrol (30μM) for 24 h.	Effect of resveratrol in ameliorating cardiac pathology in mdx mice, through mitophagy mechanism.	Mitochondrial DNA (mtDNA) deletion, autophagy of damaged mitochondria (mitophagy).	Resveratrol treatment promotes FoxO3a activity in the nucleus of cardiomyocytes compared to untreated cells.Autophagy and mitophagy are promoted by FoxOs modulation leading overall to decreased ROS accumulation in the heart [46].
6	Lecompte, S. et al. “Skeletal muscle secretomein Duchenne muscular dystrophy: a pivotal anti-inflammatory role of adiponectin”. Cell. Mol. Life Sci. (2017) 74:2487–2501	Human myotubes of dystrophic patients/human myotubes as control	Primary cultures of human myotubes treated or not with adiponectin (ApN) after an inflammatory challenge.	Role of adiponectin in Duchenne muscular dystrophy.	ApN production; levels of some pro-inflammatory molecules like (TNFα, IL-17A, and CCL28), and anti-inflammatory ones like IL6.	In dystrophic myotubes, ApN mRNA and ApN levels were decreased by ~60% and ~15%, respectively, compared to control cells. The treatment of human myotubes with ApN leads to the downregulation of pro-inflammatory markers such as TNFα (~−30%) and to the upregulation of IL-6 (~+75%) that exerts anti-inflammatory properties. The ApN pathway involves the AMPK-SIRT1-PGC-1α axis, leading to utrophin A upregulation. Overall, these data suggest that ApN activity in DMD is regulated by the AMPK-SIRT1-PGC-1α pathway [2].
7	Abou-Samra, M.A.; et al. “Involvement of adiponectin in the pathogenesis of dystrophinopathy”. Skeletal Muscle (2015) 5:25	Human myotubes	Cells were treated with human recombinant TNFα (10 ng/mL) + interferon gamma (IFNγ) (10 ng/mL) and/or ApN (5μg/mL), at the indicated concentrations, for 24 h.	Effect of adiponectin in counteracting muscle degeneration.	Effects of adiponectin on inflamed human myotubes	Adiponectin treatment after the inflammatory stimuli of TNFα and IFNγ lead to a reduction in TNFα mRNAs. This positive effect of ApN is abrogated by siRNA silencing of genes encoding for AdipoR1, SIRT1, or PGC-1α. These data suggest that the SIRT1/PGC-1α axis plays a crucial role in the anti-inflammatory activity of ApN [35].

## Data Availability

The data presented in this study are available within the article, and in the reference section.
